# Emergency Physicians' Confidence in Managing Obstetric and Gynecologic Emergencies in Saudi Arabia: A Cross-Sectional Study

**DOI:** 10.7759/cureus.106205

**Published:** 2026-03-31

**Authors:** Najla M Alsudairy, Yara K Alraddadi, Abdullah O Alahmari, Manayir F Alorabi, Shrooq A Almotairi, Abdullah S Alghamdi, Raghad N Bouges

**Affiliations:** 1 Family Medicine, National Guard Health Affairs, Jeddah, SAU; 2 Family Medicine, First Health Cluster, Jeddah, SAU; 3 General Practice, King Abdulaziz University Faculty of Medicine, Jeddah, SAU; 4 Obstetrics and Gynecology, East Jeddah General Hospital, Jeddah, SAU; 5 Obstetrics and Gynecology, King Abdullah Medical Complex, Jeddah, SAU; 6 Emergency Medicine, King Faisal Hospital, Makkah, SAU

**Keywords:** emergency department, emergency medicine, gynecologic emergencies, maternal health, obstetric emergencies, obstetric training, physician confidence, saudi arabia

## Abstract

Background: Obstetric and gynecologic emergencies are time-sensitive conditions encountered in emergency departments and may result in substantial maternal morbidity and mortality if not promptly recognized and managed. Emergency physicians are frequently the first clinicians to evaluate pregnant patients presenting with acute complications, yet exposure to obstetric emergencies and related procedural training may be limited. This study assessed the confidence of emergency physicians in Saudi Arabia in managing obstetric and gynecologic emergencies and identified factors associated with higher confidence.

Methods: We conducted a cross-sectional survey of emergency physicians practicing in Saudi Arabia between January and March 2026. A structured electronic questionnaire assessed demographic characteristics, training background, clinical exposure to obstetric emergencies, and self-reported confidence in managing specific obstetric conditions and procedures. Confidence was measured using a 5-point Likert scale (1 (not confident) to 5 (very confident)). High confidence was defined as a mean score of ≥4. Descriptive statistics were used to summarize responses, and univariable logistic regression was performed to identify factors associated with high confidence.

Results: A total of 214 emergency physicians participated. Most respondents were male (143 of 214, 66.8%) and aged 25-35 years (153 of 214, 71.5%). Formal training in obstetric emergencies during residency was reported by 132 physicians (132 of 214, 61.7%). Participants reported relatively higher confidence in managing hyperemesis gravidarum (mean score: 4.1±0.8) and first-trimester vaginal bleeding (3.9±0.9), whereas lower confidence was reported for shoulder dystocia (2.7±1.2) and emergency delivery in the emergency department (2.8±1.2). Procedural confidence was highest for neonatal resuscitation (3.8±0.9) and pelvic examination (3.6±1.0) and lowest for episiotomy and repair (2.3±1.2). Overall, 76 physicians (76 of 214, 35.5%) met the criteria for high confidence. Factors associated with higher confidence included more than five years of emergency medicine experience (odds ratio (OR): 2.41; 95% CI: 1.45-4.02), formal obstetric emergency training (OR: 2.87; 95% CI: 1.70-4.84), frequent exposure to obstetric emergencies (OR: 3.14; 95% CI: 1.82-5.43), neonatal resuscitation certification (OR: 1.68; 95% CI: 1.01-2.80), and consultant-level practice (OR: 3.56; 95% CI: 1.72-7.35).

Conclusions: Emergency physicians in Saudi Arabia reported variable confidence in managing obstetric and gynecologic emergencies, with lower confidence in complex obstetric conditions and delivery-related procedures. Greater clinical experience, formal training, and more frequent exposure to obstetric emergencies were associated with higher confidence. Targeted educational strategies, including structured obstetric emergency training and simulation-based learning, may help strengthen preparedness for time-critical obstetric emergencies in the emergency department.

## Introduction

Obstetric and gynecologic emergencies represent a critical subset of conditions encountered in emergency departments and are associated with significant maternal morbidity and mortality when not promptly recognized and managed [1,2]. Pregnant and reproductive-age women frequently present to emergency departments with acute symptoms such as abdominal pain, vaginal bleeding, and hemodynamic instability, requiring rapid assessment and timely intervention [1-3]. Emergency physicians are often the first clinicians to evaluate these patients, particularly in settings where immediate obstetric consultation may not be available [3,4]. As a result, the ability of emergency physicians to confidently recognize and manage obstetric and gynecologic emergencies plays an important role in ensuring safe and effective patient care [2-4].

Despite the importance of these skills, the frequency and complexity of obstetric emergencies in emergency departments can vary widely across institutions [3,5]. Many emergency physicians encounter such cases intermittently, which may limit opportunities to develop and maintain procedural competency in obstetric management [1-3]. Conditions such as ectopic pregnancy, postpartum hemorrhage, preeclampsia or eclampsia, and emergency delivery require not only prompt diagnosis but also familiarity with time-sensitive interventions and coordination with obstetric services [5,6]. Previous studies have suggested that gaps in training and limited clinical exposure may contribute to variability in physician confidence when managing these emergencies [3-7].

Training in obstetric and gynecologic emergencies during emergency medicine residency programs also varies between institutions and regions [2,5,8]. Although emergency physicians are expected to recognize and stabilize obstetric complications, procedural training in areas such as emergency vaginal delivery, management of shoulder dystocia, and postpartum hemorrhage may be limited [3-7]. Furthermore, in many healthcare systems, obstetric care is primarily delivered by obstetric specialists, which may further reduce hands-on exposure for emergency physicians. As a consequence, some physicians may feel less prepared to independently manage obstetric emergencies when specialist support is delayed or unavailable [2,4,5].

In Saudi Arabia, emergency medicine has expanded rapidly over the past two decades, with increasing numbers of emergency departments serving a growing and diverse patient population. However, limited data are available regarding the preparedness of emergency physicians to manage obstetric and gynecologic emergencies within this setting [2-7]. Understanding physicians' confidence levels and identifying factors associated with higher confidence may help inform training strategies and educational initiatives aimed at improving emergency care for pregnant patients.

Accordingly, this study aimed to assess the confidence of emergency physicians in Saudi Arabia in managing obstetric and gynecologic emergencies in the emergency department and to identify factors associated with higher levels of confidence.

## Materials and methods

Study design and setting

This study was a cross-sectional survey conducted to assess emergency physicians' confidence in managing obstetric and gynecologic emergencies in emergency departments across Saudi Arabia after obtaining approval from the Standing Committee for Sabbatical Leaves, Publication and Research Ethics of the Ministry of Health (approval number: REC-2025-14083). The study targeted physicians currently practicing in emergency medicine in both public and private hospitals. Data were collected over three months (January to March 2026) using a structured, self-administered questionnaire distributed electronically. Participation was voluntary, and responses were collected anonymously.

Participants and eligibility criteria

Eligible participants included emergency medicine residents, specialists, fellows, and consultants who were actively working in emergency departments in Saudi Arabia at the time of the study. Physicians practicing in other specialties, medical interns, and physicians not currently involved in emergency department clinical practice were excluded. Participation was open to physicians from different hospital types, including government tertiary hospitals, secondary hospitals, private hospitals, military hospitals, and university-affiliated institutions.

Sampling and recruitment

Participants were recruited using a convenience sampling approach. The survey link was disseminated through professional emergency medicine networks, institutional email lists, and physician communication platforms commonly used within emergency departments. Invitations were distributed through professional groups and contacts to maximize geographic representation across the central, western, eastern, northern, and southern regions of Saudi Arabia. Participants were encouraged to complete the questionnaire only once.

Survey instrument

Data were collected using a structured questionnaire developed to evaluate physician confidence in emergency and obstetric care (see Appendices). The questionnaire consisted of seven sections covering demographic characteristics, professional background, training history, clinical exposure to obstetric and gynecologic emergencies, self-reported confidence in managing specific emergencies, procedural confidence, and perceived educational needs.

Confidence in managing obstetric and gynecologic emergencies and performing related procedures was measured using a 5-point Likert scale, ranging from 1 (not confident) to 5 (very confident). The survey included items assessing confidence in the management of common emergency presentations such as ectopic pregnancy, miscarriage, preeclampsia or eclampsia, postpartum hemorrhage, placental abruption, and emergency delivery, as well as procedures including pelvic examination, point-of-care ultrasound for ectopic pregnancy, neonatal resuscitation, and episiotomy repair.

Validity and pilot testing

The questionnaire underwent content validation by three experts in emergency medicine and obstetrics, who reviewed the survey items for relevance, clarity, and completeness. Minor modifications were made based on their feedback to improve wording and ensure clinical applicability. Before full deployment, the questionnaire was pilot tested among 12 emergency physicians to evaluate clarity, feasibility, and completion time. Responses from the pilot testing were not included in the final analysis. Internal consistency of the confidence scale was assessed using Cronbach's alpha.

Outcome measures

The primary outcome was self-reported confidence in managing obstetric and gynecologic emergencies among emergency physicians. Confidence scores were calculated from the Likert-scale responses. For analytic purposes, high confidence was defined as a mean score of 4 or higher across the relevant confidence items. Secondary outcomes included procedural confidence and identification of factors associated with higher confidence levels, such as training background, years of experience, and exposure to obstetric emergencies.

Sample size calculation

The required sample size was calculated using the standard formula for estimating a proportion in cross-sectional studies. Assuming a 95% confidence level, a 5% margin of error, and an expected proportion of 50% (to provide the most conservative estimate in the absence of prior national data), the minimum required sample size was calculated to be 196 participants.

Statistical analysis

Data were analyzed using IBM SPSS Statistics for Windows, Version 27.0 (IBM Corp., Armonk, New York, United States). Descriptive statistics were used to summarize participant characteristics and survey responses. Categorical variables were reported as numbers and percentages, whereas continuous variables were presented as means with standard deviations.

Associations between physician characteristics and high confidence in managing obstetric and gynecologic emergencies were evaluated using univariable logistic regression analysis, with results reported as odds ratios with 95% confidence intervals (CI). A two-sided p-value of less than 0.05 was considered to indicate statistical significance.

## Results

Participant characteristics

A total of 214 emergency physicians completed the survey. The largest proportion of participants were 31-35 years of age (81, 37.9%), followed by 25-30 years (72, 33.6%), 36-40 years (36, 16.8%), 41-45 years (17, 7.9%), and older than 45 years (8, 3.7%). Most respondents were male (143, 66.8%), whereas 71 (33.2%) were female.

With respect to professional level, 88 participants (41.1%) were residents, 63 (29.4%) specialists, 54 (25.2%) consultants, and nine (4.2%) fellows. Regarding clinical experience, 78 physicians (36.4%) had 1-3 years of experience, 54 (25.2%) had 4-6 years, 36 (16.8%) had 7-10 years, 27 (12.6%) had more than 10 years, and 19 (8.9%) had less than one year of experience. Most respondents worked in government tertiary hospitals (109, 50.9%), followed by secondary hospitals (46, 21.5%), private hospitals (29, 13.6%), military hospitals (19, 8.9%), and university hospitals (11, 5.1%) (Table 1).

**Table 1:** Demographic and professional characteristics of the participating emergency physicians (N=214) Data are presented as number (percentage). Percentages may not total 100 because of rounding. Participants included emergency physicians practicing in emergency departments across Saudi Arabia. Professional level refers to the current clinical training or practice position of the respondent. Years of experience indicate the total time practicing in emergency medicine. Hospital type refers to the primary institution in which the participant practices. The total number of participants was 214.

Characteristic		No. (%)
Age group (years)	25-30	72 (33.6)
	31-35	81 (37.9)
	36-40	36 (16.8)
	41-45	17 (7.9)
	>45	8 (3.7)
Gender	Male	143 (66.8)
	Female	71 (33.2)
Professional level	Resident	88 (41.1)
	Specialist	63 (29.4)
	Consultant	54 (25.2)
	Fellow	9 (4.2)
Years of experience	<1 year	19 (8.9)
	1-3 years	78 (36.4)
	4-6 years	54 (25.2)
	7-10 years	36 (16.8)
	>10 years	27 (12.6)
Type of hospital	Government tertiary hospital	109 (50.9)
	Secondary hospital	46 (21.5)
	Private hospital	29 (13.6)
	Military hospital	19 (8.9)
	University hospital	11 (5.1)

Training and clinical exposure to obstetric emergencies

Overall, 132 participants (61.7%) reported receiving formal training in obstetric emergencies during residency, whereas 82 (38.3%) reported no such training. Nearly all physicians had completed Basic Life Support certification (214, 100%), and the majority had completed Advanced Cardiac Life Support (198, 92.5%) and Advanced Trauma Life Support (171, 79.9%). Fewer respondents reported training in the Neonatal Resuscitation Program (104, 48.6%), and only 37 (17.3%) had completed Advanced Life Support in Obstetrics training.

Exposure to obstetric and gynecologic emergencies varied. Ninety-seven participants (45.3%) encountered such cases weekly, 39 (18.2%) daily, 60 (28%) monthly, and 18 (8.4%) rarely. Pelvic examinations were performed occasionally by 88 physicians (41.1%), rarely by 63 (29.4%), and frequently by 36 (16.8%), whereas 27 (12.6%) reported never performing pelvic examinations. Regarding consultation availability, 92 participants (43%) reported on-site obstetrics and gynecology (OB/GYN) coverage, 105 (49.1%) reported on-call availability, and 17 (7.9%) indicated that such services were not readily available (Table 2).

**Table 2:** Training background and clinical exposure to obstetric and gynecologic emergencies among emergency physicians (N=214) Data are presented as number (percentage) of participants. Percentages may not total 100 because of rounding. Formal training refers to the structured education in obstetric emergencies received during emergency medicine residency. Completed courses indicates the certification in commonly required emergency or obstetric life-support programs. Frequency of encountering obstetric or gynecologic emergencies reflects the self-reported clinical exposure of participants in the emergency department. Pelvic examinations refer to the procedures performed by the responding physician in the emergency department. Availability of obstetrics and gynecology specialists indicates the level of access to specialist consultation at the participant's primary practice site. The total number of participants was 214. ACLS: Advanced Cardiac Life Support; BLS: Basic Life Support; ATLS: Advanced Trauma Life Support; NRP: Neonatal Resuscitation Program; ALSO: Advanced Life Support in Obstetrics; OB/GYN: obstetrics and gynecology; ED: emergency department

Variable		No. (%)
Formal training in obstetric emergencies during residency	Yes	132 (61.7)
	No	82 (38.3)
Completed courses	ACLS	198 (92.5)
	BLS	214 (100)
	ATLS	171 (79.9)
	NRP	104 (48.6)
	ALSO	37 (17.3)
Frequency of encountering OB/GYN emergencies	Daily	39 (18.2)
	Weekly	97 (45.3)
	Monthly	60 (28)
	Rarely	18 (8.4)
Pelvic examinations performed in the ED	Frequently	36 (16.8)
	Occasionally	88 (41.1)
	Rarely	63 (29.4)
	Never	27 (12.6)
Availability of OB/GYN specialists	On-site 24/7	92 (43)
	On-call	105 (49.1)
	Not available	17 (7.9)

Confidence in managing obstetric emergencies

Confidence levels varied across obstetric conditions. Participants reported relatively higher confidence in managing hyperemesis gravidarum (mean score: 4.1±0.8) and first-trimester vaginal bleeding (3.9±0.9). Moderate levels of confidence were observed for miscarriage management (3.7±0.9) and ectopic pregnancy (3.6±1.0).

Lower confidence was reported for more complex obstetric emergencies, including preeclampsia or eclampsia (3.4±1.1), postpartum hemorrhage (3.2±1.2), and placental abruption (3.1±1.1). The lowest confidence levels were observed for emergency delivery in the emergency department (2.8±1.2) and shoulder dystocia (2.7±1.2) (Table 3).

**Table 3:** Emergency physicians' self-reported confidence in managing selected obstetric emergencies (N=214) Confidence was assessed using a 5-point Likert scale ranging from 1 (not confident) to 5 (very confident). Values are presented as mean±standard deviation. Higher scores indicate greater self-reported confidence in managing the specified obstetric condition in the emergency department. Responses reflect physicians' perceived confidence rather than objective measures of clinical competence. ED: emergency department

Obstetric emergency	Mean confidence score±SD
First-trimester vaginal bleeding	3.9±0.9
Ectopic pregnancy	3.6±1.0
Miscarriage management	3.7±0.9
Hyperemesis gravidarum	4.1±0.8
Preeclampsia/eclampsia	3.4±1.1
Postpartum hemorrhage	3.2±1.2
Placental abruption	3.1±1.1
Preterm labor assessment	3.0±1.1
Shoulder dystocia	2.7±1.2
Emergency delivery in the ED	2.8±1.2

Confidence in performing obstetric procedures

Participants demonstrated varying degrees of confidence in performing OB/GYN-related procedures. The highest procedural confidence was reported for neonatal resuscitation (3.8±0.9) and pelvic examination (3.6±1.0). Moderate confidence was reported for point-of-care ultrasound for ectopic pregnancy (3.1±1.1) and management of postpartum hemorrhage (3.0±1.1).

Lower confidence levels were noted for transvaginal ultrasound interpretation (2.9±1.2) and vaginal delivery in the emergency department (2.7±1.3), whereas the lowest confidence score was observed for episiotomy and repair (2.3±1.2) (Table 4).

**Table 4:** Emergency physicians' self-reported confidence in performing obstetric and gynecologic procedures (N=214) Confidence was assessed using a 5-point Likert scale ranging from 1 (not confident) to 5 (very confident). Values are presented as mean±standard deviation. Higher scores indicate greater self-reported confidence in performing the specified procedure in the emergency department setting. Responses reflect perceived confidence rather than objective procedural competency. ED: emergency department

Procedure	Mean confidence score±SD
Pelvic examination	3.6±1.0
Transvaginal ultrasound interpretation	2.9±1.2
Point-of-care ultrasound for ectopic pregnancy	3.1±1.1
Vaginal delivery in the ED	2.7±1.3
Episiotomy and repair	2.3±1.2
Management of postpartum hemorrhage	3.0±1.1
Neonatal resuscitation	3.8±0.9

Factors associated with high confidence

Overall, 76 participants (35.5%) met the criteria for high confidence in managing OB/GYN emergencies. Physicians with more than five years of emergency medicine experience (58, 51.3%) had significantly higher odds of reporting high confidence than those with less experience (odds ratio: 2.41; 95% CI: 1.45-4.02; p<0.001).

Similarly, respondents who had received formal obstetric emergency training during residency (76, 57.6%) were more likely to report high confidence compared with those without such training (odds ratio: 2.87; 95% CI: 1.70-4.84; p<0.001). Frequent exposure to obstetric emergencies was also associated with higher confidence, with 63 physicians (61.2%) reporting high confidence among those encountering such cases regularly (odds ratio: 3.14; 95% CI: 1.82-5.43; p<0.001).

In addition, NRP-certified physicians (49, 47.1%) had modestly higher confidence compared with those without certification (odds ratio: 1.68; 95% CI: 1.01-2.80; p=0.045). Confidence was also higher among consultants (38, 70.4%), who demonstrated greater odds of reporting high confidence compared with physicians at earlier career stages (odds ratio: 3.56; 95% CI: 1.72-7.35; p<0.001) (Table 5).

**Table 5:** Factors associated with high confidence in managing obstetric and gynecologic emergencies among emergency physicians High confidence was defined as a mean confidence score of ≥4 across the obstetric emergency management items measured on a 5-point Likert scale (range: 1-5; higher scores indicate greater confidence). Values in the "High confidence n (%)" column represent the number and percentage of participants within each subgroup who met the high-confidence threshold; percentages were calculated using the total number of participants in each respective category as the denominator. Odds ratios with 95% CI were estimated using the logistic regression analysis. An asterisk (*) indicates statistical significance (p<0.05). CI: confidence interval; NRP: Neonatal Resuscitation Program

Variable	High confidence n (%)	Odds ratio (95% CI)	P-value
>5 years of experience	58 (51.3)	2.41 (1.45-4.02)	<0.001*
Formal obstetric emergency training	76 (57.6)	2.87 (1.70-4.84)	<0.001*
Frequent exposure to obstetric emergencies	63 (61.2)	3.14 (1.82-5.43)	<0.001*
NRP certification	49 (47.1)	1.68 (1.01-2.80)	0.045*
Consultant level	38 (70.4)	3.56 (1.72-7.35)	<0.001*

## Discussion

In this cross-sectional survey of 214 emergency physicians practicing in Saudi Arabia, we found that overall confidence in managing obstetric and gynecologic emergencies varied considerably depending on the clinical condition and required procedural skills. Emergency physicians reported relatively higher confidence in managing common and medically oriented conditions such as hyperemesis gravidarum, miscarriage, and first-trimester vaginal bleeding, whereas confidence was substantially lower for complex obstetric emergencies requiring procedural or delivery-related skills, including shoulder dystocia, emergency delivery in the emergency department, and episiotomy repair. In addition, greater clinical experience, formal obstetric emergency training, frequent exposure to obstetric cases, and neonatal resuscitation certification were associated with higher levels of confidence.

The findings highlight an important pattern in emergency medicine training. Physicians generally reported stronger confidence in conditions that involve diagnostic assessment and medical management, while procedures that are traditionally performed in obstetric settings were associated with lower confidence levels. These findings are consistent with the structure of emergency medicine practice in many hospitals, where obstetric care is often managed primarily by OB/GYN specialists [5-10]. As a result, emergency physicians may have limited opportunities to independently perform delivery-related procedures, which may contribute to lower confidence levels in these areas.

The association between clinical experience and physician confidence observed in this study is consistent with the broader literature on clinical competency in emergency medicine [11-14]. Physicians with more than five years of experience were significantly more likely to report higher confidence levels in managing obstetric emergencies. Increased experience likely reflects greater exposure to diverse clinical presentations and repeated opportunities to participate in multidisciplinary obstetric care. Similarly, frequent exposure to obstetric emergencies in the emergency department was associated with higher confidence, suggesting that clinical volume and case exposure play an important role in developing competency.

Another notable finding was the impact of formal training in obstetric emergencies. Participants who reported receiving structured obstetric emergency training during residency were significantly more likely to report higher confidence levels. Despite this association, fewer than two-thirds of physicians in this study reported having received such training. Moreover, only a small proportion of participants had completed specialized obstetric life-support courses. These findings suggest that although emergency physicians regularly encounter obstetric emergencies, formal training opportunities may remain limited, particularly in structured simulation or obstetric emergency courses [13-16].

Procedural confidence also varied substantially. Participants reported moderate confidence in performing pelvic examinations and neonatal resuscitation, but substantially lower confidence in performing procedures such as transvaginal ultrasound interpretation, vaginal delivery in the emergency department, and episiotomy repair. The relatively higher confidence in neonatal resuscitation may reflect the widespread implementation of neonatal resuscitation training programs, which are commonly incorporated into emergency medicine and pediatric training curricula. In contrast, procedural obstetric skills are often less emphasized in emergency medicine training programs, which may explain the lower reported confidence in these areas.

These findings have important implications for emergency medicine education and workforce preparedness. Obstetric emergencies, although relatively infrequent compared with other emergency presentations, can be time-sensitive and associated with significant maternal and neonatal morbidity. Emergency physicians are often the first clinicians to evaluate pregnant patients presenting with acute complications, particularly in settings where obstetric services are not immediately available. Ensuring that emergency physicians maintain adequate competency in recognizing and stabilizing obstetric emergencies is therefore an important component of emergency care systems.

Several strategies may help address the gaps identified in this study. Simulation-based training programs, interdisciplinary obstetric emergency drills, and structured obstetric rotations during emergency medicine residency may help improve physician confidence and procedural competency. In addition, continuing medical education programs focused on obstetric emergencies may provide opportunities for practicing emergency physicians to maintain and enhance relevant clinical skills.

This study has several limitations. First, the findings are based on self-reported confidence, which may not directly reflect actual clinical competence or performance in real-world settings. Second, the use of a convenience sampling approach may introduce selection bias, and physicians with a greater interest in obstetric emergencies may have been more likely to participate. Third, although efforts were made to include participants from different regions and hospital types, the sample may not fully represent all emergency physicians practicing in Saudi Arabia. Finally, the cross-sectional design of the study precludes conclusions regarding causality between training factors and physician confidence.

## Conclusions

In this cross-sectional survey of emergency physicians practicing in Saudi Arabia, confidence in managing obstetric and gynecologic emergencies varied substantially across clinical conditions and procedures. Physicians reported relatively higher confidence in the management of common obstetric presentations but lower confidence in complex obstetric emergencies and delivery-related procedures. Greater clinical experience, formal obstetric emergency training, and more frequent exposure to obstetric cases were associated with higher levels of confidence. These findings suggest that targeted educational strategies, including structured obstetric emergency training and simulation-based learning, may help strengthen emergency physicians' preparedness to manage time-critical obstetric emergencies in the emergency department.

## Appendices

**Table 6:** Survey questionnaire administered to emergency physicians The questionnaire assessed demographic characteristics, professional background, training history, clinical exposure to obstetric and gynecologic emergencies, self-reported confidence in managing specific emergencies and performing related procedures, and perceived educational needs. Confidence items were measured on a 5-point Likert scale, where 1 means not confident and 5 means very confident. Response options for categorical items are shown in the table. ACLS: Advanced Cardiac Life Support; BLS: Basic Life Support; ATLS: Advanced Trauma Life Support; NRP: Neonatal Resuscitation Program; ALSO: Advanced Life Support in Obstetrics; ED: emergency department; OB/GYN: obstetrics and gynecology

Section	Question/item	Response options/scale
Demographics	Age	25-30, 31-35, 36-40, 41-45, >45 years
	Gender	Male, female
	Professional level	Resident, specialist, fellow, consultant
	Years of experience in emergency medicine	<1, 1-3, 4-6, 7-10, >10 years
	Type of hospital	Government tertiary, secondary, private, military, university
Training history	Did you receive formal training in obstetric emergencies during residency?	Yes, no
	Completed courses	BLS, ACLS, ATLS, NRP, ALSO
Clinical exposure	How often do you encounter obstetric and gynecologic emergencies in the ED?	Daily, weekly, monthly, rarely
	How often do you perform pelvic examinations in the ED?	Frequently, occasionally, rarely, never
	Availability of OB/GYN specialists in your hospital	On-site 24/7, on-call, not available
Confidence in managing obstetric emergencies	First-trimester vaginal bleeding	1 (not confident) to 5 (very confident)
	Ectopic pregnancy	1-5 Likert scale
	Miscarriage management	1-5 Likert scale
	Hyperemesis gravidarum	1-5 Likert scale
	Preeclampsia/eclampsia	1-5 Likert scale
	Postpartum hemorrhage	1-5 Likert scale
	Placental abruption	1-5 Likert scale
	Preterm labor assessment	1-5 Likert scale
	Shoulder dystocia	1-5 Likert scale
	Emergency delivery in the ED	1-5 Likert scale
Confidence in performing procedures	Pelvic examination	1-5 Likert scale
	Transvaginal ultrasound interpretation	1-5 Likert scale
	Point-of-care ultrasound for ectopic pregnancy	1-5 Likert scale
	Vaginal delivery in the ED	1-5 Likert scale
	Episiotomy and repair	1-5 Likert scale
	Management of postpartum hemorrhage	1-5 Likert scale
	Neonatal resuscitation	1-5 Likert scale
Perceived educational needs	Do you feel additional training in obstetric emergencies is needed?	Yes, no
	Preferred method for additional training	Simulation-based training, workshops, online modules, other (specify)

## References

[1] Mandell MG, Avila B, Baker KA (2025). Assessment of gynecologic emergency triage and appointment wait times: a mystery caller study. Cureus.

[2] Mitzman J, Pfeil SA, Rahurkar S (2025). Virtual obstetric emergency simulations: enhancing knowledge, skills, and confidence of emergency medicine and obstetric professionals. Am J Perinatol.

[3] Agarwal I, Sarma D, Burke RC (2023). Resident attitudes toward performing pelvic examinations in the emergency department. Womens Health Rep (New Rochelle).

[4] Raoust GM, Bergström J, Bolin M, Hansson SR (2022). Decision-making during obstetric emergencies: a narrative approach. PLoS One.

[5] Govender S, Khaliq OP, Abel T, Moodley J (2025). Knowledge, attitude and practice of emergency care providers on obstetric haemorrhage in KwaZulu-Natal, South Africa: a cross-sectional study. Afr J Emerg Med.

[6] Høgh S, Thellesen L, Bergholt T, Rom AL, Johansen M, Sorensen JL (2021). How often will midwives and obstetricians experience obstetric emergencies or high-risk deliveries: a national cross-sectional study. BMJ Open.

[7] Casper S, Kayani T, Galerneau F, Evans L, Pal L (2023). Improving preparedness of emergency medicine residents in the management of postpartum hemorrhage: a randomized controlled study of two pedagogical approaches. Int J Gynaecol Obstet.

[8] Johanson RB, Menon V, Burns E (2002). Managing Obstetric Emergencies and Trauma (MOET) structured skills training in Armenia, utilising models and reality based scenarios. BMC Med Educ.

[9] Musie MR, Tagutanazvo OB, Sepeng NV, Mulaudzi FM, Hlongwane T (2025). A scoping review on continuing professional development programs for midwives: optimising management of obstetric emergencies and complications. BMC Med Educ.

[10] Ameh CA, Mdegela M, White S, van den Broek N (2019). The effectiveness of training in emergency obstetric care: a systematic literature review. Health Policy Plan.

[11] Myers G, Huckaby A, Buderer N (2025). Improving comfort in obstetric skills of emergency medicine residents with lecture- and simulation-based training. Cureus.

[12] Mahgoub EA, Osman SH, Al-Hussien HA, Al-Bushra N, Khairy A, Elhadi YA (2024). Examining the use, confidence, and barriers to follow Advanced Life Support in Obstetrics (ALSO) course guides in managing obstetric emergencies in Sudan. BMC Med Educ.

[13] Nieto-Gutierrez W, Taype-Rondan A (2023). Self-perceived competence in managing obstetric emergencies among recently graduated physicians from Lima, Peru. BMC Med Educ.

[14] Shah N, Shah N, Rabab UE, Tariq H, Ayaz S, Fahim T (2024). Assessing emergency room leadership of ObGyn residents in a public university teaching hospital of Sindh, Pakistan: a cross-sectional survey. BMC Med Educ.

[15] Janicki AJ, MacKuen C, Hauspurg A, Cohn J (2016). Obstetric training in emergency medicine: a needs assessment. Med Educ Online.

[16] Mahalekshmi KP, S R, T S M, Ms S (2025). Evaluation of knowledge, attitude, and practices of compulsory rotating medical interns in managing obstetric emergencies in a tertiary hospital. Cureus.

